# Publisher Correction: Multi-Modal Mobility Morphobot (M4) with appendage repurposing for locomotion plasticity enhancement

**DOI:** 10.1038/s41467-023-40466-9

**Published:** 2023-08-07

**Authors:** Eric Sihite, Arash Kalantari, Reza Nemovi, Alireza Ramezani, Morteza Gharib

**Affiliations:** 1 Aerospace Engineering Department, California Institute of Technology, 1200 E California Blvd, Pasadena, CA USA; 2 Jet Propulsion Laboratory (JPL), 4800 Oak Grove Drive, M/S 82-105 Pasadena, CA USA; 3 Electrical and Computer Engineering Department, Northeastern University, 360 Huntington Ave, Boston, MA USA

**Keywords:** Aerospace engineering, Biomimetics

Correction to: *Nature Communications* 10.1038/s41467-023-39018-y, published online 27 June 2023

The original version of this Article omitted a reference to previous work in ‘Meiri, N. et al. Flying STAR, a hybrid crawling and flying sprawl tuned robot, In 2019 IEEE International Conference on Robotics and Automation (ICRA), 5302–5308 (IEEEE, 2019).’ This has been added as reference 44 in the sixth line of the seventh paragraph of the introduction: “However, M4 differs from refs. 42, 43, 44 work because M4 exhaust appendage redundancy manipulation through morphing to maximize locomotion plasticity.” This has been corrected in the PDF and HTML versions of the Article.

The Peer Review File associated with this Article was updated shortly after publication to remove the mistakenly copied article file.

### Supplementary information

Updated Peer Review file

