# Supplementary material for: Multi-Modal Mobility Morphobot (M4) with appendage repurposing for locomotion plasticity enhancement
Source: Nat Commun. 2023 Jun 27;14:3323. doi: 10.1038/s41467-023-39018-y (PMC10300070; doi:10.1038/s41467-023-39018-y)
Supplement: Supplementary file 1 — Supplementary Information [file 41467_2023_39018_MOESM1_ESM.pdf]

Supplementary Information: Multi-Modal Mobility Morphobot (M4) with Appendage Repurposing for Locomotion Plasticity Enhancement

Eric Sihite [1], Arash Kalantari [2], Reza Nemovi [1], Alireza Ramezani [3*], and Morteza Gharib [1]

[1]Aerospace Engineering Department, California Institute of Technology, 1200 E California Blvd, Pasadena, California, USA.

[2]Jet Propulsion Laboratory (JPL), 4800 Oak Grove Drive, M/S 82-105, Pasadena, California, USA

[3]Electrical and Computer Engineering Department, Northeastern University, 360 Huntington Ave, Boston, Massachusetts, USA

*Corresponding author email: a.ramezani@northeastern.edu

Supplementary Notes

This supplementary note includes three composite figures referenced in the main draft. These figures illustrate:

1- A conceptual design of a machine with extensive locomotion plasticity (Supplementary Figure 1).
2- Simulated multi-modal path planning results based on multi-modal PRM (Supplementary Figure 2).
3- WAIR simulation trajectory (Supplementary Figure 3).

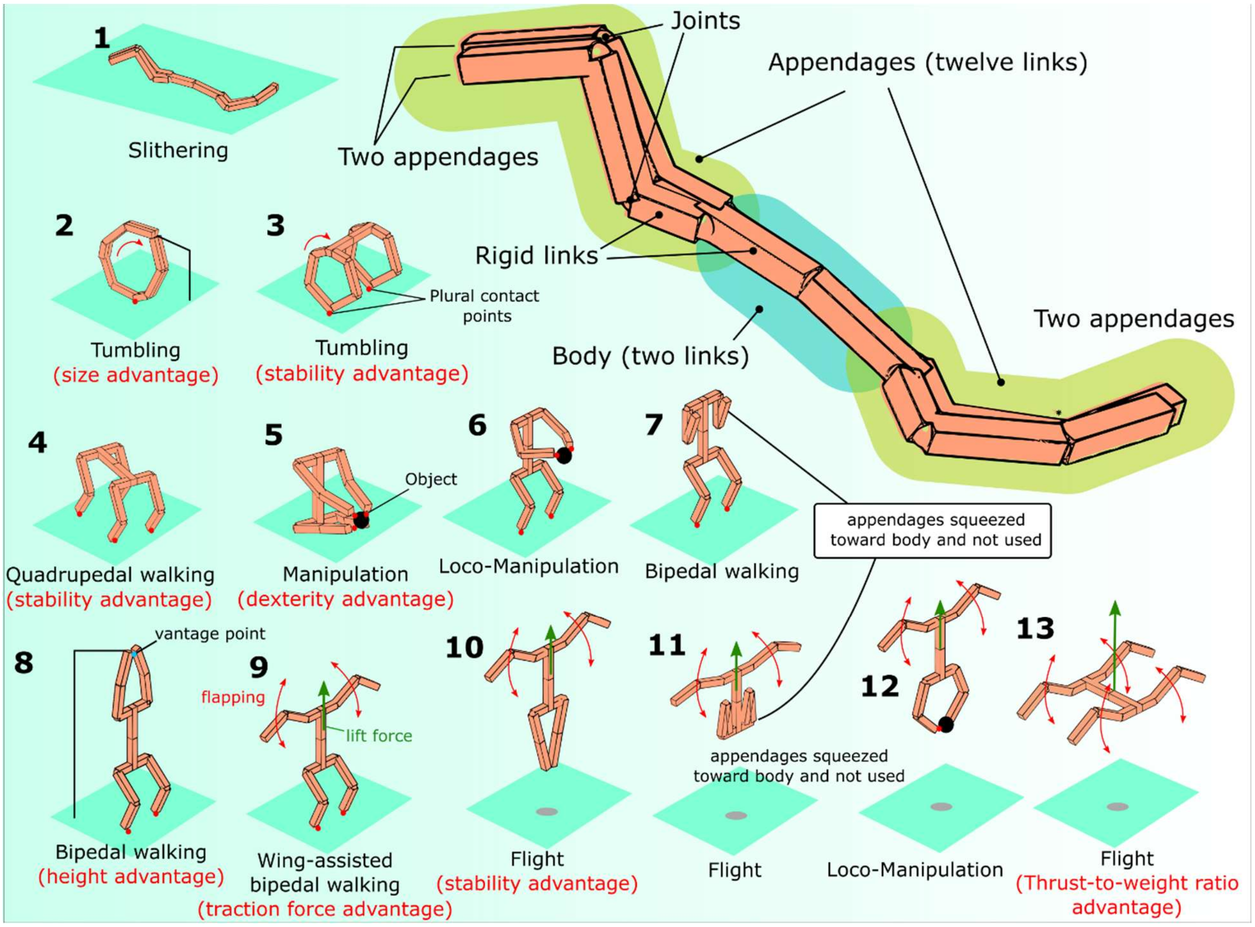

Supplementary Figure 1: Shows an imaginary morpho-functional robot, where body morphing and appendage repurposing are utilized to achieve several modes. This concept is not physically realizable due to large range of motions in joints, part collisions, and actuation challenges. It is only used for illustration purposes. Note that within each numbered state, other modes of operation are conceivable (e.g., walking, trotting, galloping, etc., within mode #4), yielding additional locomotion plasticity.

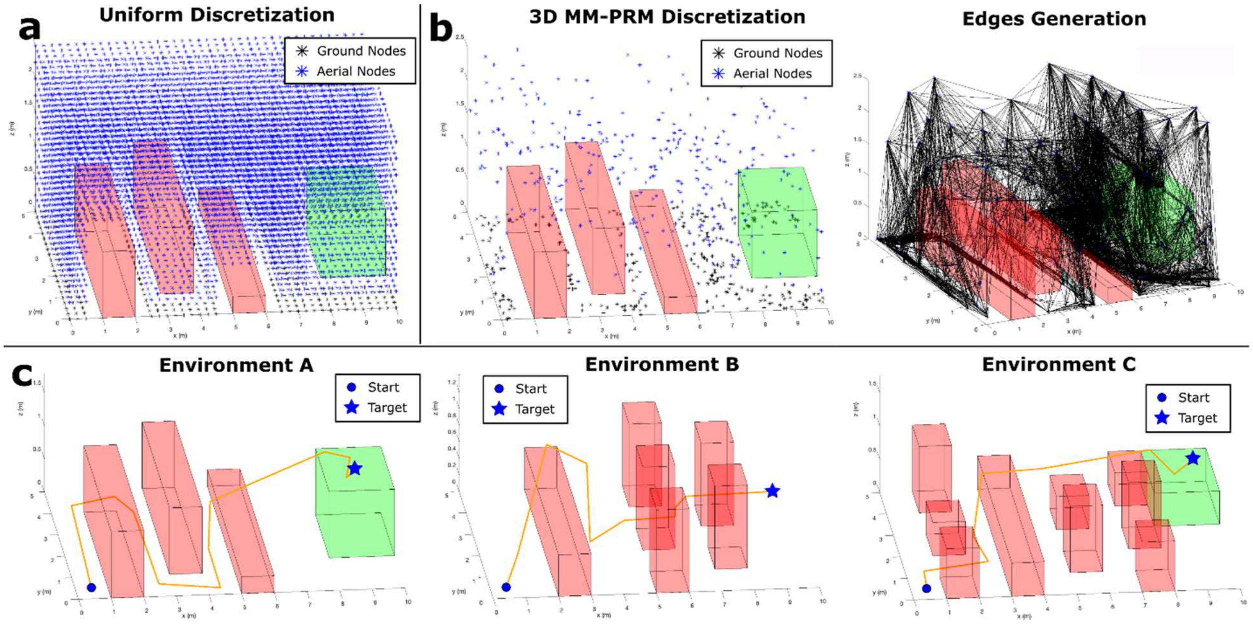

Supplementary Figure 2: Illustration of the path planning algorithm for navigating an environment using both ground and aerial mobility. **(a)** Representation of the set of nodes generated by a uniform grid discretization. **(b)** Example of graph generated by the 3D MM-PRM Algorithm with the following parameters: *R* = 4 meters, *Nw* = 300, and *Nf* = 300. The MM-RPM method generates a significantly reduced amount of nodes which greatly reduces the computational time and cost of performing the path-finding algorithm. **(c)** The trajectories generated by the 3D A * path planning algorithm on three different environments. The red box represents an obstacle that cannot be driven over, while the green box represents a drive-able platform.

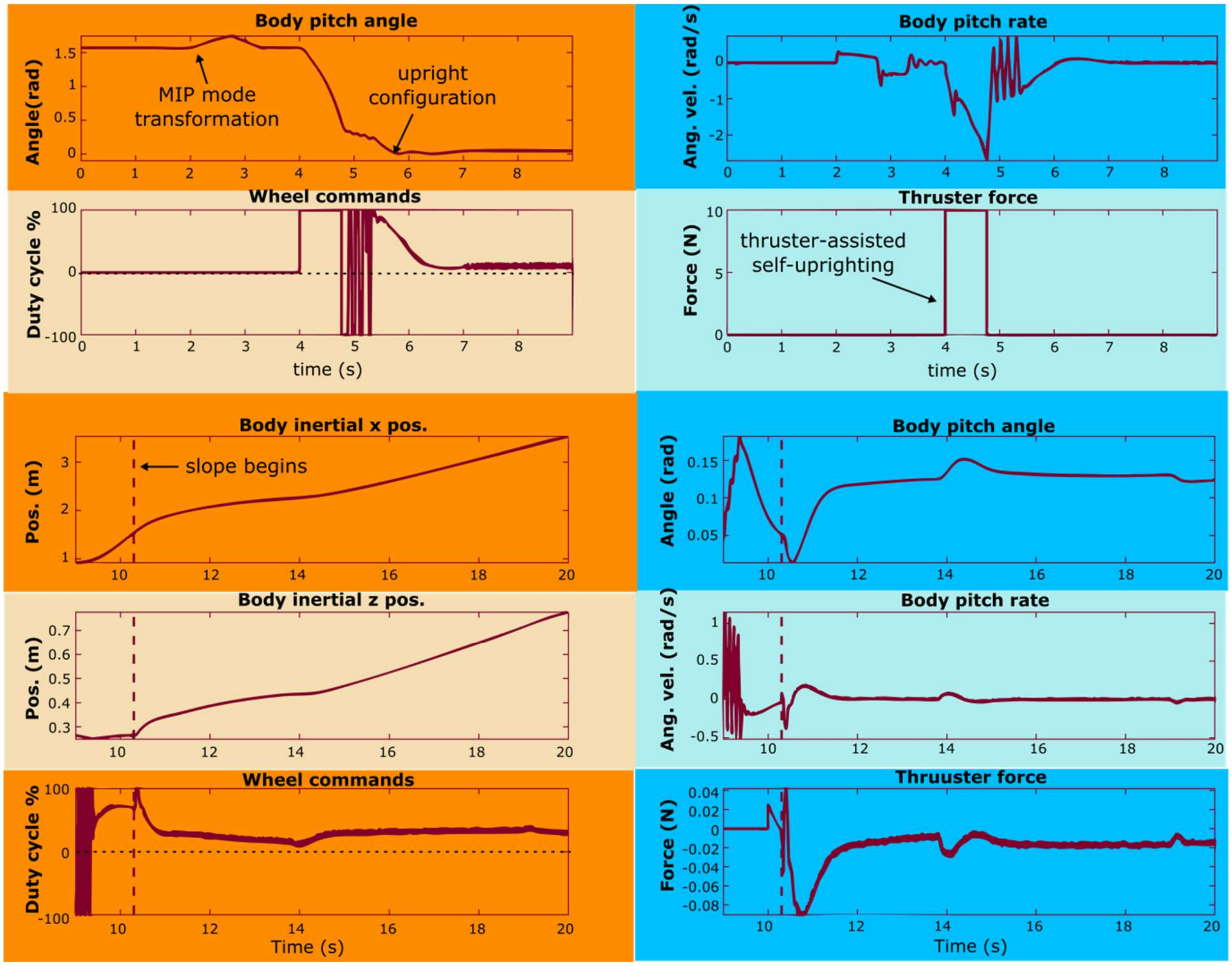

Supplementary Figure 3: Plots in the first two rows show the pitch angle states and controller inputs during the thruster-assisted self-uprighting maneuver. Plots in rows 3, 4, and 5 depicts the states and controller inputs during WAIR. The non-zero thruster force on the slope indicates that the thrusters assisted in driving up the incline.
