# Supplementary material for: Multi-Modal Mobility Morphobot (M4) with appendage repurposing for locomotion plasticity enhancement
Source: Nat Commun. 2023 Jun 27;14:3323. doi: 10.1038/s41467-023-39018-y (PMC10300070; doi:10.1038/s41467-023-39018-y)
Supplement: Supplementary file 3 — Description of Additional Supplementary Files [file 41467_2023_39018_MOESM3_ESM.pdf]

File Name: Supplementary Video 1

Description:

A series of videos of the robot performing crouching locomotion to pass through a low-height ceiling, loco-manipulation to grasp an object using its appendages, and legged locomotion to walk over rough terrain.

File Name: Supplementary Video 2

Description:

A series of videos showing the robot driving in rover mode and transforming into UAV mode to fly over a pond.

File Name: Supplementary Video 3

Description:

A video showing the robot performing "wing-assisted incline running (WAIR)", where the robot in upright MIP configuration uses its upper thrusters to climb on a steep slope that the robot is unable to climb with other ground locomotion modes. This maneuver is performed on 30- and 45-deg slopes.

File Name: Supplementary Video 4

Description:

A video showing the robot performing a tumbling maneuver to vault over an obstacle that cannot be driven over. The robot transforms its front propellers to face upwards and lifts the upper body up while the rear wheels drive the robot forward to vault over the obstacles. Then, the same sequence is performed for the rear side and the robot transforms back into UGV once the obstacle has been cleared.

File Name: Supplementary Video 5

Description:

A video showing the robot transforming from UGV into upright MIP configuration, and vice-versa. The robot utilizes the upper thrusters to pitch up or down during the transition.

File Name: Supplementary Video 6

Description:

A video showing the robot using its multi-modal capability to autonomously follow a set of waypoints with a motion capture system in the loop. The robot starts in UGV mode, drives near the tower, transforms into UAV mode, flies and lands on top of the tower, then transforms back to UGV mode.

File Name: Supplementary Video 7

Description:

A video showing the robot autonomously navigating a complex environment with SLAM using an onboard stereo-depth camera and computer. The target waypoint is provided by the user, then the robot calculates a ground or aerial path to the target point while exploiting M4's multi-modal capability.
